# Salmonid gene expression biomarkers indicative of physiological responses to changes in salinity and temperature, but not dissolved oxygen

**DOI:** 10.1242/jeb.198036

**Published:** 2019-07-05

**Authors:** Aimee Lee S. Houde, Arash Akbarzadeh, Oliver P. Günther, Shaorong Li, David A. Patterson, Anthony P. Farrell, Scott G. Hinch, Kristina M. Miller

**Affiliations:** 1 Department of Forest and Conservation Sciences, University of British Columbia, Vancouver, BC, Canada, V6T 1Z4; 2 Pacific Biological Station, Fisheries and Oceans Canada, Nanaimo, BC, Canada, V9T 6N7; 3 Department of Fisheries, Faculty of Marine Science and Technology, University of Hormozgan, PO Box 3995, Bandar Abbas, Iran; 4 Günther Analytics, 402-5775 Hampton Place, Vancouver, BC, Canada, V6T 2G6; 5 School of Resource and Environmental Management, Fisheries and Oceans Canada, Simon Fraser University, Burnaby, BC, Canada, V5A 1S6; 6 Department of Zoology and Faculty of Land and Food Systems, University of British Columbia, Vancouver, BC, Canada, V6T 1Z4

**Keywords:** Transcription, Environmental stressors, Smolt status, Na^+^/K^+^-ATPase activity, Plasma ions, Behaviour

## Abstract

An organism's ability to respond effectively to environmental change is critical to its survival. Yet, life stage and overall condition can dictate tolerance thresholds to heightened environmental stressors, such that stress may not be equally felt across individuals and at all times. Also, the transcriptional responses induced by environmental changes can reflect both generalized responses as well as others that are highly specific to the type of change being experienced. Thus, if transcriptional biomarkers specific to a stressor, even under multi-stressor conditions, can be identified, the biomarkers could then be applied in natural environments to determine when and where an individual experiences such a stressor. Here, we experimentally challenged juvenile Chinook salmon (*Oncorhynchus tshawytscha*) to validate candidate gill gene expression biomarkers. A sophisticated experimental design manipulated salinity (freshwater, brackish water and seawater), temperature (10, 14 and 18°C) and dissolved oxygen (normoxia and hypoxia) in all 18 possible combinations for 6 days using separate trials for three smolt statuses (pre-smolt, smolt and de-smolt). In addition, changes in juvenile behaviour, plasma variables, gill Na^+^/K^+^-ATPase activity, body size, body morphology and skin pigmentation supplemented the gene expression responses. We identified biomarkers specific to salinity and temperature that transcended the multiple stressors, smolt status and mortality (live, dead and moribund). Similar biomarkers for dissolved oxygen were not identified. This work demonstrates the unique power of gene expression biomarkers to identify a specific stressor even under multi-stressor conditions, and we discuss our next steps for hypoxia biomarkers using an RNA-seq study.

## Abstract (precis)

**Summary:** Gene expression biomarkers associated with salinity stress and thermal stress, but not hypoxic stress, exist in salmon gill tissue. Biomarkers were also associated with mortality.

## INTRODUCTION

Declining wild populations in multitudes of species worldwide have been associated with environmental change, suggesting that physiological limits of tolerance are being reached (Wikelski and Cooke, 2006). Notably, the early marine survival of Pacific salmon juveniles from many populations in southern British Columbia has diminished rapidly over the past 20 years, with hatchery smolt releases typically suffering the lowest survival rates (Beamish et al., 2009; Bradford and Irvine, 2000; Martins et al., 2012; Riddell et al., 2013). Factors contributing to this particular decline are beginning to emerge. For example, annual variability in the marine survival is associated, in part, with climate regime shifts, which can result in periods of heightened environmental stressors, including higher water temperatures and reduced water dissolved oxygen (DO) (Crozier, 2016). Although environmental monitoring provides information on the potential for heightened stressors, the degree to which an animal is actually exposed to these stressors is typically lacking. Therefore, examining individual salmon can be a more direct and integrative approach to assess the magnitude of different stressor impacts (Wikelski and Cooke, 2006). For example, panels of gene expression biomarkers associated with transcriptional responses have been associated with specific environmental stressors (Connon et al., 2018). Indeed, a biomarker approach has already successfully examined the relationship of viral disease status and survival in the natural environment among Pacific salmon species (Miller et al., 2017a). In the present study, our aim was to experimentally validate such biomarkers during the smoltification period of a salmon, a particularly critical development period, and overlay the interactive effects of salinity, temperature and hypoxia (low DO) challenges.

During smoltification, juvenile anadromous salmonids physiologically prepare for the transition from freshwater to seawater. Inadequate smoltification or ill-timed seawater entry outside of their physiological seawater tolerance window (i.e. as pre-smolts or de-smolts) can cause more than 40% mortality or stunted growth for 1–2 months (Stien et al., 2013), as well as well-documented physiological disturbances. Furthermore, hatchery-produced juveniles experience a larger physiological stress during their freshwater to seawater transition relative to wild juveniles, which has been associated, in part, with reduced survival (e.g. Chittenden et al., 2008; Shrimpton et al., 1994). Also, the smoltification window narrows at higher water temperatures (Bassett et al., 2018), and even adult salmon can similarly experience a salinity stress if they are physiologically unprepared when returning to rivers (Cooke et al., 2006). Although climate change is contributing to lower DO and higher temperature, the impact of hypoxia on smoltification is unknown.

Critical thresholds for detrimental impacts for most populations of salmonids are DO <6 mg l^−1^ and temperature >18°C (Lucas and Southgate, 2003). Thermal stress is associated with decreased survival and fitness-related traits (e.g. swim performance, body growth and disease resistance) across all salmonid life stages (Quinn et al., 2011; Tomalty et al., 2015), although the effects on juveniles are much less studied. Furthermore, billions of juvenile salmon annually experience hypoxia as they pass through estuaries on the North American Pacific coast because of excessive nutrient loading, lower water discharge and the increased prevalence of algal blooms (Birtwell and Kruzynski, 1989). Eutrophic lakes can also become hypoxic (Conley et al., 2009), which may be a concern because smoltification can begin in lakes, e.g. Sockeye salmon in Cultus Lake, British Columbia (Putt, 2014). Hypoxic stress, owing to repeated or prolonged exposure to low DO, is detrimental for fish activity, feeding, growth rates and other normal biological functions (Closs et al., 2016).

As a non-lethal tool that uses a gill tissue biopsy for measuring gene expression, we have already discovered 93 candidate genes for salinity (*n=*37; Houde et al., 2018 preprint), temperature (*n*=33; Akbarzadeh et al., 2018) and DO (*n*=23; Table S1) for all Pacific salmon species. The candidate salinity genes, originally developed to broadly measure the degree of smoltification (Houde et al., 2018 preprint), are specifically used in the present study to examine whether experimental manipulation of salinity will directly influence the regulation of some of these genes. Similarly, the candidate temperature genes, which were previously discovered for a single stressor (Akbarzadeh et al., 2018), are considered in the present study under multi-stressor conditions. The candidate DO genes were derived primarily from a literature review of a few transcriptomic studies on hypoxia response. Besides our candidate genes, there are validated biomarkers already for viral disease development (Miller et al., 2017a), smoltification (Houde et al., 2018 preprint), as well as general stress and imminent mortality (Evans et al., 2011; Jeffries et al., 2012, 2014a,b). These earlier studies, like the present one, used the Fluidigm BioMark™ platform, a high throughput microfluidics technology that can independently measure the gene expression of 96 assays by 96 samples at once. This approach also has been used to assess pathogen loads (Miller et al., 2016). Ultimately, the aim is to produce a salmon ‘Fit-Chip’, potentially the first tool of its kind, using a suite of biomarkers that would comprehensively assess the physiological health and condition of cultured and wild fish.

Our first objective was to identify the biomarkers specific to the salinity, temperature and DO treatments that transcended these multi-stressor conditions. Also, smolt status (pre-smolt, smolt and de-smolt) and mortality (live, dead and moribund fish) were considered because fish collection in the natural environment may include individuals that are dead or moribund and of uncertain smoltification stage. Besides, the expression patterns among live and moribund fish are already known to be similar for a number of our candidate genes (e.g. temperature, Akbarzadeh et al., 2018). Therefore, ocean-type Chinook salmon (*Oncorhynchus tshawytscha*) were experimentally challenged with three salinities, three temperatures and two DO values in all 18 possible combinations for 6 days using four trials that spanned their smoltification period between March and August (i.e. pre-smolt, smolt and de-smolt). For the trials with pre-smolts and de-smolts, some mortality was expected in seawater. Therefore, a second objective was to identify biomarkers specifically associated with both mortality and the physiological imbalance associated with poorly timed seawater entry. The biomarker association and body traits known for smoltification have been reported elsewhere, using the same candidate salinity genes (Houde et al., 2018 preprint). Given that expression patterns of the candidate temperature genes were similar for juvenile and adult salmonids (Akbarzadeh et al., 2018), we had no smolt status expectations for temperature and hypoxia.

Few studies have specifically documented the association between gene expression and other physiological measures or fitness-related traits important to the conservation of fishes (e.g. Connon et al., 2018; Oomen and Hutchings, 2017). Hence, beyond measurements of mortality, we also collected measures of salmon fitness-related traits, i.e. behaviour, skin pigmentation and body morphology, as well as physiological biomarkers of stress and seawater tolerance, i.e. plasma lactate, glucose and chloride concentrations (Barton, 2002), and gill Na^+^/K^+^ ATPase (NKA) activity (McCormick, 1993). These variables were then associated with the gene expression patterns and differences among groups within treatments.

## MATERIALS AND METHODS

### Study species

The experiment was approved by the Fisheries and Oceans (DFO) Pacific Region Animal Care Committee (2017-002 and 2017-020) which abides by the Canadian Council of Animal Care Standards. Sub-yearling ocean-type Chinook salmon [*Oncorhynchus tshawytscha* (Walbaum 1792)] were transported from Big Qualicum River Hatchery, Qualicum Beach, British Columbia (BC), Canada, to the Pacific Biological Station, Nanaimo, BC, on 15 and 29 May 2017 and 26 February 2018. For transport, fish were sedated with Aquacalm (0.1 mg l^−1^, Syndel, Nanaimo, BC, Canada) within containers that had portable aerators and Vidalife mucous protectant (15 ml l^−1^, Syndel). Fish were reared in communal circular tanks supplied with dechlorinated municipal freshwater (10 to 14°C), aeration, and artificial light set at the natural cycle until they were used in the experimental trials. They were fed a 2% body mass ration of pellets (Bio-Oregon, Vancouver, BC, Canada) every 1‒2 days.

### Experimental set-up

**Fig. 1.**
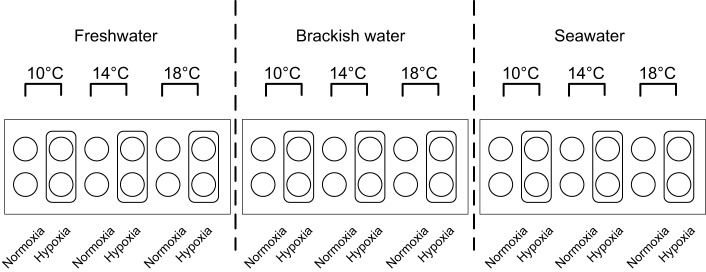
**Experimental design and tank layout of the 18 test groups for the three treatments: three salinities, three temperatures and two dissolved oxygen concentrations.**

The fish were divided into 18 test groups (Fig. 1), composed of all possible combinations of treatments for salinity (freshwater at 0 PSU, brackish water at 20 PSU and seawater at 28 or 29 PSU), temperature (10, 14 and 18°C) and DO (hypoxia at 4‒5 mg l^−1^ and normoxia at >8 mg l^−1^). Each group was represented by duplicate 30 liter pot tanks with tight fitting lids that limited gas exchange. Seawater was pumped from nearby Departure Bay and disinfected using ultraviolet light. Both freshwater and seawater were provided at ambient temperatures, as well as chilled and heated, for a total of six available water sources. The experimental salinities and temperatures were achieved using combinations of these water sources; the brackish groups were produced using a two-step process of water passing through a mixer to achieve 20 PSU that was then divided into metal coils within three water baths to achieve the temperatures. Two columns, one containing plastic media (1.91–3.81 cm bio-rings) for aeration and the other containing a ceramic air stone to introduce very small (5–10 μm) nitrogen bubbles from either portable liquid units or compressed gas bottles (Praxair, Nanaimo, BC, Canada), regulated the water DO. For one of all duplicate hypoxic tanks, probes continuously monitored temperature and DO to maintain DO within 4‒5 mg l^−1^ by turning the nitrogen regulator on or off as required through individual Point Four RIU3monitor-controllers (Pentair, Minneapolis, MN, USA) that were connected to a Point Four LC3central water system (Pentair). Water was gravity fed from the bottom of the columns to the experimental tanks using 1.27 cm internal diameter PVC pipe or tubing. Additional technical details on the experimental set-up are available from the authors.

### Trial protocol and fish welfare

Four independent trials spanned the pre-smolt stage (trial 1), through the smolt stage (trial 2), to the de-smolt stage (trials 3 and 4) (Table 1). These smolt statuses were confirmed by the expected differences in skin pigmentation, body morphology, NKA activity and seawater mortality (see Results). All fish in the communal tank were starved for 24 or 48 h, sedated with Aquacalm and the water was treated with Vidalife. Individual fish were haphazardly selected, lightly anaesthetized in buffered TMS (100 mg l^−1^, Syndel), measured for fork length (±0.1 cm) and mass (±0.01 g), digitally photographed (Nikon Coolpix AW110, Minato, Tokyo, Japan) on their left side alongside size and colour standards for later skin pigmentation and body morphology measurements. Fish were then recovered with aeration before being moved to an experimental tank for a 6-day habituation period. Surfaces in contact with juveniles were sprayed liberally with concentrated Vidalife and care was taken so that juveniles were not out of water for more than 30 s. Each tank contained either 12 (in 2017) or 16 (in 2018) fish and was supplied with freshwater (13‒14°C and >8 mg l^−1^ DO) during the 6-day habituation period, during which they were fed a 2% body mass ration per day.

**Table 1.**
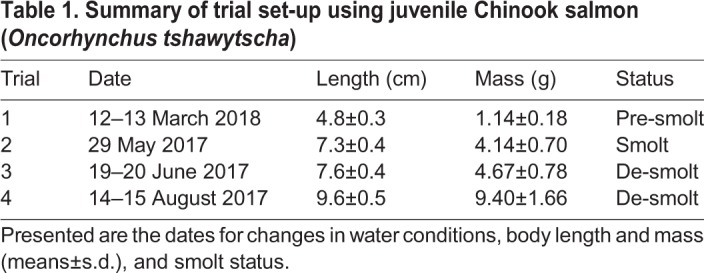
**Summary of trial set-up using juvenile Chinook salmon (*Oncorhynchus tshawytscha*)**

Prior to establishing the test water conditions, fish were starved for 24 or 48 h. Seawater was introduced over 2‒3 h in the morning on day 1. Temperature was changed by 2°C in the early afternoon of day 1 and by a further 2°C on day 2. DO was set to 6.5‒8 mg l^−1^ at 15:00 h on day 1 and to 4‒5 mg l^−1^ in the morning of day 2. Feeding restarted in the morning of day 3. Test exposures then lasted 6 days, during which fish welfare was visually inspected at least every 4 h between 08:00 and 20:00 h. During each inspection, any dead juveniles (no gill ventilation) were removed and moribund juveniles (loss of equilibrium, but gill ventilation) were euthanized with an overdose of TMS (250 mg l^−1^, buffered for freshwater groups) using water of the same salinity and temperature as the test conditions.

### Fish sampling

Short-term gill gene expression was monitored on day 2 of the test exposure. Fish were either anaesthetized (4 of 12 fish per tank in 2017) or euthanized (4 of 16 fish per tank in 2018) for measurements and photographs as described above. Anaesthetized fish were placed on their back in a cushioned trough under lamp light and then a small piece of gill tissue (1×1 mm) was removed from the tip of filaments using sterilized scissors. A visible elastomer implant (Northwest Marine Technology, Shaw Island, WA, USA) was inserted below the dorsal fin. The fish was then revived in aerated water of the same salinity and temperature as the test conditions before being returned to their tank. Fish were never out of aerated water for more than 30 s. Surfaces in contact with juveniles were sprayed liberally with Vidalife. Tools were disinfected between each fish using 3‒5 min of 10% bleach and immersion in 95% ethanol and flame, and the tools were cooled before use with the next fish. All gill tissue samples were individually placed in RNAlater (Invitrogen, Carlsbad, CA, USA) for 24 h in a 4°C fridge and then stored in a −80°C freezer until used for gene expression measurements.

Behavioural observations were made on fish between 11:00 and15:00 h on day 4 of the test exposure, i.e. after they had received their morning ration. A trained observer made behavioural observations for 3 min for each tank in trial 2 (May 2017) with the tank cover removed. The observer recorded the relative startle response (i.e. swimming speed after disturbance) and gill ventilation rate. For subsequent trials (1, 3 and 4; March 2018, June 2017 and August 2017), 10-min video clips were collected for each tank using small cameras held in an underwater case (GoPro Hero, San Mateo, CA, USA or Monster Digital Villain, Simi Valley, CA, USA). Raw video clips were brightened and trimmed (removing first and last 30 s) using GoPro Studio 2.5. Edited video clips were renamed to hide the tank identity and scored by two trained observers, who calculated the gill ventilation rate for three fish per tank.

All remaining fish were euthanized, as described above, on day 6 of the test exposure. Fish were measured and photographed (Nikon Coolpix AW110 or Olympus Tough TG-3, Shinjuku, Tokyo, Japan). Blood was collected into a capillary tube within 5 min by severing caudal vessels, then centrifuged (2000 ***g***) for 5 min to separate plasma, which was then immediately frozen using dry ice. For gill NKA activity measurements, gill tissue was removed from the right side, placed into a cryovial and then immediately frozen using liquid nitrogen. For examining long-term gene expression, gill tissue from the left side was placed in RNAlater, as described above. The rest of the body was individually placed into a plastic bag and frozen immediately with dry ice. All tissues were stored in a −80°C freezer until used for measurements.

### Gene expression

Gill gene expression was examined in 441 fish samples. These fish samples included two to three haphazardly selected live individuals from each tank for each of the four trials (316 juveniles) that had received a 6-day test exposure, as well as 125 dead or moribund individuals (mostly pre-smolts and de-smolts from trials 1, 3 and 4). Our detailed analysis focused on the 6-day gill samples because the expression of a subset of the 2-day gill samples (representing 33 fish of trial 1) was generally similar to that of the 6-day samples (see Data availability).

Gill tissue was homogenized in TRIzol (Ambion, Foster City, CA, USA) and BCP reagent (Sigma-Aldrich, Oakville, ON, Canada) using stainless steel beads on an MM301 mixer mill (Retsch, Haan, Germany). RNA was extracted from the homogenate using the ‘No-Spin Procedure’ of MagMAX-96 Total RNA Isolation kits (Ambion) and a Biomek FXP automation workstation (Beckman Coulter, Mississauga, ON, Canada). RNA yield was quantified using the *A*_260_ value and extracts were normalized to 62.5 ng ml^−1^. Normalized RNA was reverse transcribed to cDNA using SuperScript VILO synthesis kits (Invitrogen). Normalized RNA and cDNA were stored at −80°C between steps.

Gene expression was examined for three housekeeping genes, i.e. *Coil-P84*, *78d16.1* and *MrpL40* (Miller et al., 2017a), and 93 candidate genes, i.e. 37 for salinity, 33 for temperature and 23 for DO. Each gene expression chip contained these 96 assays, serial dilutions of a cDNA pool (1, 1/5, 1/25, 1/125, 1/625 and 1/3125) and an inter-chip calibrator sample. Following Fluidigm (South San Francisco, CA, USA) prescribed methods, target cDNA sequences were enriched using the specific target amplification (STA) method that included small concentrations of the 96 assay primer pairs. Specifically, for each reaction, this included 3.76 μl 1X TaqMan PreAmp master mix (Applied Biosystems, Foster City, CA, USA), 0.2 μmol l^−1^ of each of the primers and 1.24 μl of cDNA. Samples were run on a 14-cycle PCR program, with excess primers removed with EXO-SAP-IT (Affymetrix, Santa Clara, CA, USA), and diluted 1/5 in DNA suspension buffer. The diluted samples and assays were run in singleton following the Fluidigm platform instructions. Sample reactions contained 3.0 μl 2X TaqMan mastermix (Thermo Fisher Scientific, Ottawa, ON, Canada), 0.3 μl 20X GE sample loading reagent (Fluidigm) and 2.7 μl STA product. Assay reactions contained 3.3 μl 2X assay loading reagent (Fluidigm), 0.7 μl DNA suspension buffer, 1.08 μl forward and reverse primers (50 μmol l^−1^) and 1.2 μl probe (10 μmol l^−1^). The PCR was 50°C for 2 min, 95°C for 10 min, followed by 40 cycles of 95°C for 15 s, and then 60°C for 1 min. Data were extracted using the Real-Time PCR Analysis Software (Fluidigm) with *C*_t_ set manually for each assay.

For optimal normalization, gene expression of the three housekeeping genes was first linearly transformed (efficiency^minimum *C*_t_–sample *C*_t_^), then the values were used to identify the gene or gene pair with the best stability (lowest standard deviation) using the NormFinder R function (Andersen et al., 2004) with assemblages for the 18 groups. Sample gene expression was normalized with the ΔΔ*C*_t_ method (Livak and Schmittgen, 2001) using the mean (for single gene) or geometric mean (for a pair of genes) and the calibrator sample. Gene expression was then log transformed: log_2_(2^–ΔΔ*C*_t_^).

Eighty-seven out of 93 candidate genes were used in analyses. The gene assays for *glu_2*, *LDH_3*, *Myo_1* (DO) and *CIRBP_10* (temperature) were removed because of poor efficiency using the present study samples. Also removed were *Tuba1a_16* (temperature), because the assay did not work for half of the samples, and *HIF1A_4* (DO), because this assay was not represented for smolts in trial 2 and there was no pattern with the treatment for the remaining trials (data not shown).

### NKA activity, plasma variables and infectious agents

All individuals used for gene expression analysis were examined for gill NKA activity (McCormick, 1993). Plasma lactate, glucose and chloride concentrations were measured (Farrell et al., 2001) in a subset of individuals from trials 2, 3 and 4. Although plasma cortisol is associated with stress, we did not examine this hormone because it is naturally elevated during seawater acclimation alone (McCormick, 2001; Young et al., 1995), which confounds data interpretations.

The presence and load of 47 infectious agents known or suspected to cause diseases in salmon were examined (Miller et al., 2016) in a mixed-tissue sample (gill, liver, heart, kidney and spleen) for 79 dead or moribund juveniles from trials 2, 3 and 4. Pathogen loads (with >1000 copies per μg RNA) for the 79 dead or moribund fish were limited, as only two detections out of 47 candidate pathogens in only a few individuals. The pathogens were: bacteria *Candidatus Branchiomonas cysticola* and *Flavobacterium psychrophilum* (data not shown).

### Skin pigmentation and body morphology

Skin pigmentation and body morphology was determined from photographs (Houde et al., 2015) in subset of individuals: half from the initial set-up and half after the 6-day test exposure (including all those examined for gene expression for each trial). Briefly, for skin pigmentation, LAB colour space values of the anterior region, posterior region (both covering the lateral line) and caudal fin were subjected to principal component analysis (PCA). For body morphology, 21 landmarks were subjected to relative warp analysis using tpsRelw32 software (https://life.bio.sunysb.edu/morph/soft-tps.html). Body condition was calculated as 100×mass/length^3^ (Fulton, 1904).

### Statistical analysis of variables

Analyses were performed using R 3.3.3 (R Core Team) at a significance level of α=0.05. To identify any potential treatment effects, the expression of each gene was subjected to forward model selection using Akaike's information criteria, including salinity, temperature and oxygen with their interactions. Mortality (proportion of dead and euthanized moribund juveniles) per tank was examined using binomial generalized linear models with a quasi link. The significance of effects for mortality was examined using analysis of deviance (ANODEV) *F*-tests and ANOVA for remaining variables. Tukey's *post hoc* tests examined the significance of contrasts. Gill ventilation rate, NKA activity, plasma, body size, skin pigmentation and body morphology variables were examined for simple Pearson correlations with gene expression patterns (PC1 and PC2) described below.

### Statistical analysis to identify biomarkers

We targeted clusters containing eight to 12 genes (biomarkers) that collectively co-varied in response to the salinity, temperature and DO treatments because such clusters are more likely to be conserved across species, as was found for viral disease development (Miller et al., 2017a). To this end, the entire dataset was divided into an analytical training set (two-thirds) and an analytical testing set (one-third). The training set was subjected to supervised gene shaving of the candidate genes for each treatment (Hastie et al., 2000) using GeneClust JS (Do et al., 2003). We selected the first, the first two and the first three clusters to be in range of eight to 12 identified biomarkers. Two validation approaches were implemented. First, identified genes were subjected to PCA for the training set. This PCA was then applied to the testing set to visualize unsupervised group separation within a treatment using the fviz_pca function of the factoextra R package (https://cran.r-project.org/web/packages/factoextra/index.html). This function provided 95% confidence ellipses for groups within the training set. Second, classification ability of the groups was examined in the training set by subjecting the identified biomarkers to a linear discriminant analysis (LDA), followed by a determination of classification performance on the testing set. A similar approach was adopted to examine the effect of mortality (dead or moribund versus live) using all the candidate genes.

## RESULTS

### Fish mortality

In trial 3 with de-smolts, we discovered that the DO sensor was set at a freshwater setting, causing DO to be lower than that intended for seawater, which resulted in 11 of 24 fish being dead or moribund within 2 days for the seawater–18°C–hypoxia tanks. Therefore, this group was terminated and the remaining 13 juveniles were ethically euthanized. This group was then restarted with new fish 10 days later than the original start date and the data were included in the analysis of trial 3.

Mortality data were analyzed separately for non-biopsied and biopsied juveniles because overall mortality was 2.3 times higher for fish receiving a gill biopsy after a 2-day test exposure compared with non-biopsied fish (ANODEV, *P=*0.003; Table 2). As expected for smolt status in seawater, overall mortality for non-biopsied fish was significantly lower (Tukey's *post hoc* test, *P<*0.005 for all) for smolts relative to pre-smolts and de-smolts. In particular, 11 of the 16 pre-smolts in trial 1 were dead or moribund in a seawater–18°C–hypoxia tank before this tank was terminated and the remaining five fish were euthanized. Thus, the mortality of non-biopsied juveniles was associated with salinity for trials 1, 3 and 4 based on model selection (ANODEV, *P<*0.001 for all) and mortality was significantly higher for fish held in seawater than in either freshwater or brackish water (Tukey, *P*<0.016).

**Table 2.**
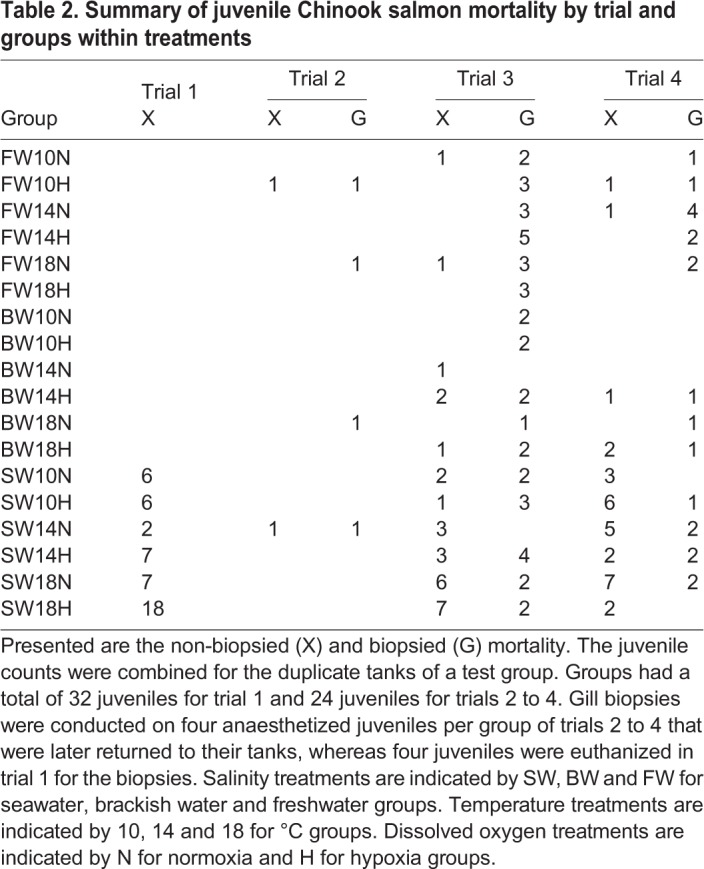
**Summary of juvenile Chinook salmon mortality by trial and groups within treatments**

Fish mortality in the trials with a biopsy differed significantly among trials (Tukey, *P*<0.005), suggesting an effect of smolt status (smolt and de-smolt; Table 2). Gill biopsies were not taken for pre-smolts in trial 1. Mortality was also associated with DO (trial 3; ANODEV, *P=*0.043) with higher mortality in hypoxia than in normoxia.

### Forward model selection

Table 3 presents forward model selection results on live fish. The gene expression data for the 87 candidate genes remaining after quality assurance checks are available from a public repository (see Data availability). Salinity primarily influenced the gene expression of 20 of the 37 candidate salinity genes; temperature influenced 10 of these 20 genes. Temperature primarily influenced the expression of 25 of the 31 candidate temperature genes; salinity influenced 19 of these 25 genes. Oxygen primarily influenced just one (i.e. *HIF1A_6*) of the 19 candidate DO genes. Oxygen followed after salinity and temperature, influencing three specific genes (i.e. *ALD_1*, *Enolase_2* and *HemA1_1*). By including the candidate temperature genes, oxygen primarily influenced one gene (*COX6B1_19*) and one other gene after temperature and salinity (*HSP90alike_6*). The specific biomarkers associated with each treatment under multi-stressor conditions and smolt status are described below.

**Table 3.**
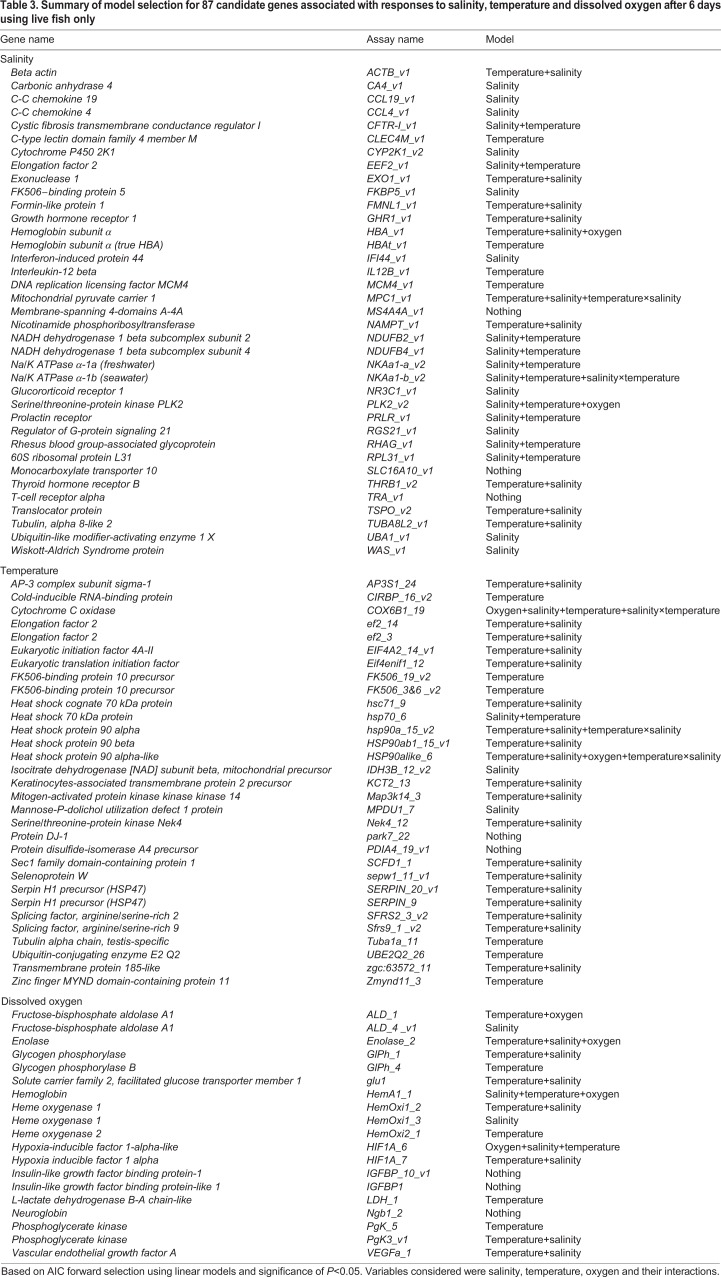
**Summary of model selection for 87 candidate genes associated with responses to salinity, temperature and dissolved oxygen after 6** **days using live fish only**

### Salinity biomarkers

**Fig. 2.**
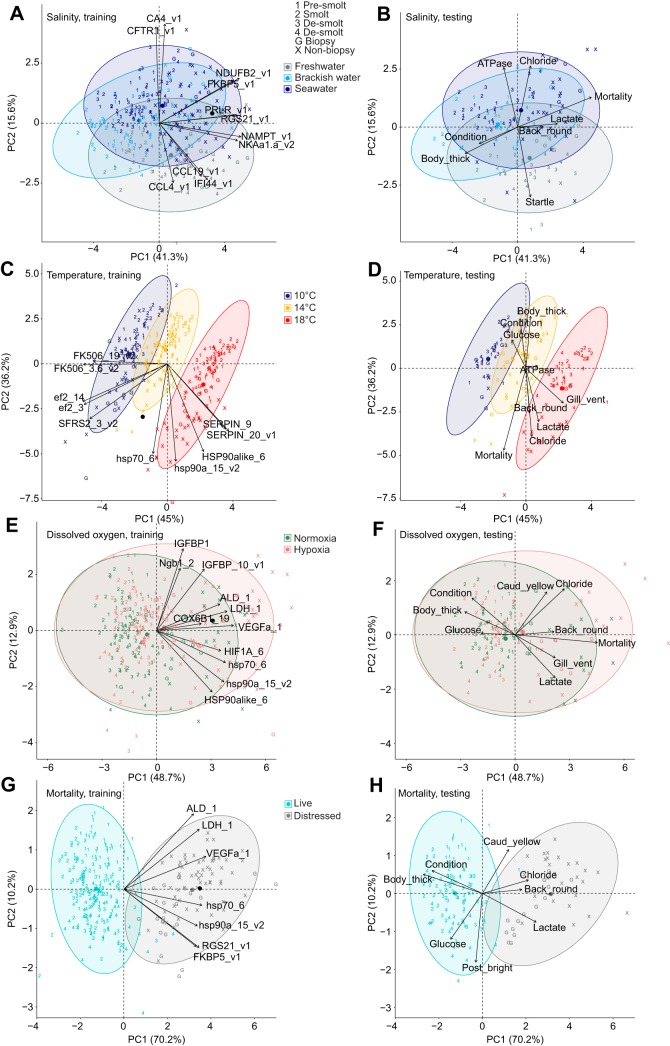
**Canonical plots of the first two principal components (PCs) of the identified biomarkers for salinity, temperature, dissolved oxygen and mortality using live and distressed fish.** (A–H) Principal component analysis was performed on the training set (left panel) and then applied to the testing set (right panel). Training set sample sizes are: *n*=82 for freshwater, *n*=73 for brackish water, *n*=139 for seawater, *n*=97 for 10°C, *n*=91 for 14°C, *n*=106 for 18°C, *n*=131 for normoxia and *n*=163 for hypoxia. Testing set sample sizes are: *n*=43 for freshwater, *n*=35 for brackish water, *n*=69 for seawater, *n*=43 for 10°C, *n*=51 for 14°C, *n*=53 for 18°C, *n*=68 for normoxia and *n*=79 for hypoxia. Ellipses represent 95% confidence areas for the groups within treatments using the training set; centroids are represented by the largest point of the same colour. Numbers are live juveniles, and X and G are dead or moribund juveniles, respectively. Arrows (left panel) represent loading vectors of the biomarkers using the training set; the arrow centroid is a black circle. Arrows (right panels) represent loading vectors of variables using the subset of available data across the training and testing sets. Variable correlations with gene expression are presented in Table S3.

Salinity biomarkers had two clusters, totalling 11 genes, using live and distressed (moribund or dead) fish. Cluster 1 contained *CA4* and *CFTR-I*, while cluster 2 contained *CCL19*, *CCL4*, *FKBP5*, *IFI44*, *NAMPT*, *NDUFB2*, *NKAa1-a*, *PRLR* and *RGS21* (Fig. 2A,B). The PCA for the training set revealed that very few dead or moribund fish fell outside the 95% confidence ellipses. Separation existed for the salinity groups along PC2, with freshwater separating from brackish water and seawater, unlike brackish water and seawater. Mortality and smolt status were better associated with PC1, with smolt at one extreme and mortality at the other, while pre-smolt and de-smolt were in between. The direction of mortality in seawater was associated with higher expression of *FKBP5*, *NKAa1-a*, *PRLR* and *RGS21*, as well as decreased expression of *CFTR-I*. When all 11 biomarkers for the training set were used in LDA, classification ability for freshwater was 100%, while seawater was 81% and brackish water was 57% (Table 4). Furthermore, when the seawater and brackish water groups were combined, classification ability was almost perfect (99%).

**Table 4.**
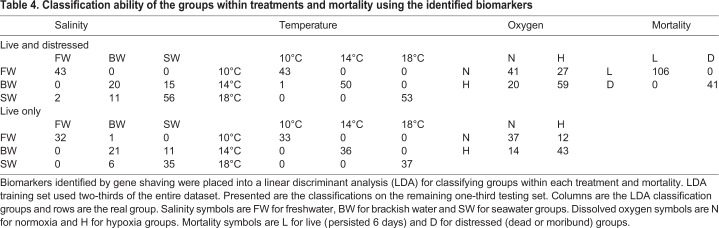
**Classification ability of the groups within treatments and mortality using the identified biomarkers**

**Fig. 3.**
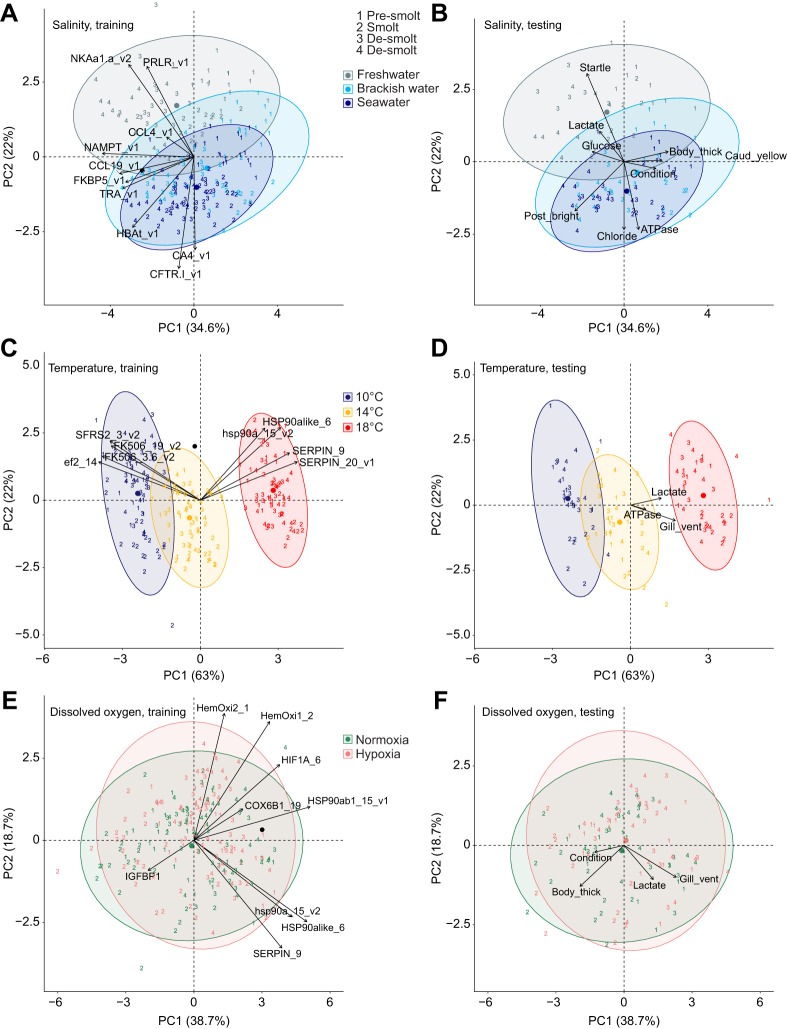
**Canonical plots of the first two principal components of the identified biomarkers for salinity, temperature and dissolved oxygen using live fish only.** (A–F) Training set sample sizes are: *n*=63 for freshwater, *n*=64 for brackish water, *n*=83 for seawater, *n*=71 for 10°C, *n*=68 for 14°C, *n*=71 for 18°C, *n*=99 for normoxia and *n*=111 for hypoxia. Testing set sample sizes are: *n*=33 for freshwater, *n*=32 for brackish water, *n*=41 for seawater, *n*=33 for 10°C, *n*=36 for 14°C, *n*=37 for 18°C, *n*=49 for normoxia and *n*=57 for hypoxia. See Fig. 2 legend for additional details.

Salinity biomarkers had two clusters with 10 genes using live fish. The genes of cluster 1 (*CA4* and *CFTR-I*) and six genes of cluster 2 (*CCL19*, *CCL4*, *FKBP5*, *NAMPT*, *NKAa1-a* and *PRLR*) were the same as those using both live and distressed fish. Cluster 2 also contained *HBAt* and *TRA* using live fish, but not *IFI44*, *NDUFB2* and *RGS21* using live and distressed fish (Fig. 3A,B). PC2 continued to separate freshwater and saline (brackish water and seawater) groups. Although PC1 also represented smolt status, smolt was now in between, with pre-smolt and de-smolt at the extremes. Classification ability increased by 9% for brackish water (66%) and decreased slightly (<4% difference) for freshwater and seawater (Table 4). Classification ability was also almost perfect (99%) when seawater and brackish water groups were combined.

### Temperature biomarkers

Temperature biomarkers had three clusters, totalling 10 genes, using live and distressed fish. Cluster 1 contained *HSP90alike6*, *SERPIN_20* and *SERPIN_9*, while cluster 2 contained *ef2_14*, *ef2_3*, *hsp70_6*, *hsp90a_15* and *SFRS2_3*. Cluster 3 contained *FK506_19* and *FK506_3.6* (Fig. 2C,D). The PCA separated the temperature groups with little overlap of the 95% confidence ellipses, and few dead or moribund individuals fell outside of these ellipses. The highest temperature (18°C) was easily separated from the cooler temperatures (10 and 14°C). Classification ability approached perfection for all three temperatures (the lowest was 98% for 14°C) (Table 4).

Temperature biomarkers had three clusters with eight genes using live fish only. The genes of cluster 1 (*HSP90alike6*, *SERPIN_20* and *SERPIN_9*) were the same as those for both live and distressed fish. Across clusters 2 and 3, there were no new genes, but *ef2_3* and *hsp70_6* were not significant contributors to the separation of groups (Fig. 3C,D). The highest temperature (18°C) continued to be easily separated from the cooler temperatures (10 and 14°C). Classification ability was 100% for the temperature groups (Table 4).

### DO biomarkers

DO biomarkers had two clusters, totalling 11 genes, using live and distressed fish. Because forward model selection indicated issues when normoxia and hypoxia were separated using only the candidate DO genes, this analysis considered all the candidate temperature and DO genes. Cluster 1 contained *ALD_1*, *COX6B1_19*, *HIF1A_6*, *hsp70_6*, *IGFBP_10*, *IGFBP1* and *LDH_1*, while cluster 2 contained *hsp90a_15*, *HSP90alike_6*, *Ngb1_2* and *VEGFa_1* (Fig. 2E,F). Regardless, normoxia and hypoxia were poorly separated by PCA. Consequently, classification ability was lower for normoxia (60%) and hypoxia (75%) (Table 4).

DO biomarkers had two clusters with nine genes using only live fish. Approximately half of the biomarkers (five genes) were the same as when using both live and distressed fish: *COX6B1_19* and *HIF1A_6* (cluster 1), as well as *hsp90a_15*, *HSP90alike_6* and *IGFBP1* (cluster 2). Remaining for cluster 2 was *HemOxi1_2*, *HemOxi2_1*, *HSP90ab1_15* and *SERPIN_9* (Fig. 3E,F). Normoxia and hypoxia continued to be poorly separated by PCA. Yet, using live fish only, classification ability increased by 16% for normoxia (76%) while there was no change for hypoxia (75%) (Table 4). Separate analyses by smolt status revealed no appreciable increase in classification ability (data not shown).

### Classification across treatments

Across all 18 test groups using the biomarkers of live and distressed fish, the average classification ability for all the three treatments was 55%, but primary classification issues existed for normoxia and hypoxia (Table S2). Removing the DO treatment (regrouping as nine salinity by temperature groups), the average classification ability increased to 80%, with secondary classification issues for brackish water and seawater. Combining brackish water with seawater, the average classification ability reached a remarkable 98%.

Although the classification issues were similar using the biomarkers of the live fish only, the classification ability was slightly increased relative to the biomarkers of live and distressed fish (Table S2). Specifically, classification ability increased by 7% across the 18 groups (62%), 3% for the nine groups (83%) and no change for the six groups (98%).

### Mortality gene expression

Dead and moribund juveniles generally had a similar gene expression profile (see Data availability). Therefore, they were combined and termed as mortality for additional analysis. Mortality across all 18 groups had a single cluster, totalling seven genes: *ALD_1*, *FKBP5*, *hsp70_6*, *hsp90a_15*, *LDH_1*, *RGS21* and *VEGFa_1* (Fig. 2G,H). The PCA ellipses had little overlap and few individuals falling outside the ellipses. A good separation for live fish and distressed (dead or moribund) fish existed, with a classification ability of 100% (Table 4).

### Differences among body variables and NKA activity

We explored the first four skin pigmentation principal components (PCs) and first four body morphology relative warp (RW) axes that were biologically meaningful. Skin pigmentation was represented by anterior brightness (PC1, 45.8%), caudal fin darkness (PC2, 25.0%), posterior brightness (PC3, 13.7%) and caudal fin yellowness (PC4, 9.7%), while body morphology was represented by body elongation or distance between fins (RW1, 19.6%), back roundness (RW2, 12.4%), caudal peduncle length (RW4, 8.4%) and body thickness or distance of the mid-section from top to bottom (RW5, 5.9%). RW3 (11.5%) was not considered because it represented body flexing during anaesthesia.

**Fig. 4.**
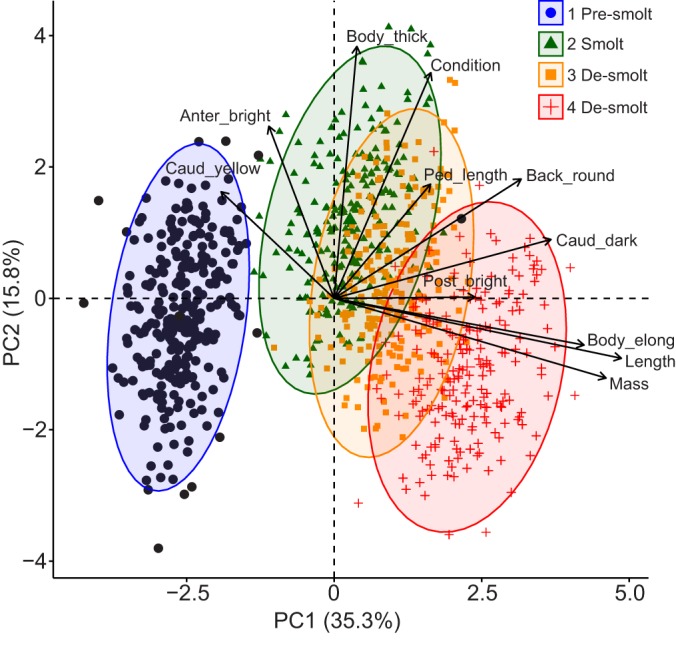
**Canonical plots of the first two principal components of initial body size, skin pigmentation and body morphology variables for the four trials.** Trials are presented in the order of smoltification, and smolt statuses are based on seawater survival. Sample sizes are: *n*=548 for pre-smolt in trial 1, *n*=432 for smolt in trial 2, *n*=474 for de-smolt in trial 3 and *n*=469 for de-smolt in trial 4. Ellipses represent 95% confidence areas for the trials; centroids are represented by the largest point of the same colour. Arrows represent loading vectors of the body traits; the arrow centroid is a black circle.

Body variables for all trials differed significantly relative to initial values (ANOVA, *P*<0.001 for all; data not shown). Not surprisingly, body length and mass increased with time (Fig. 4). As expected, differences in skin pigmentation and body morphology existed across trials according to smolt status. For example, brightness (anterior and posterior) as well as darkness and yellowness (caudal fin) increased from pre-smolts (trial 1) to smolts (trial 2). Then, brightness (anterior) and yellowness (caudal fin) decreased for de-smolts (trials 3 and 4), with smaller changes for darkness (caudal fin) and brightness (posterior). Also, body condition, elongation, thickness, back roundness and caudal peduncle length increased from pre-smolts (trial 1) to smolts (trial 2), then body thickness decreased for de-smolts (trials 3 and 4), with smaller changes for the remaining variables.

As expected, NKA activity of smolts in freshwater (trial 2) was higher than pre-smolts in freshwater (trial 1; one-tailed Student's *t*-test, *P*=0.037; Table 5). However, overall smolt NKA activity was not significantly different compared with de-smolt NKA activity (trial 3 *P*=0.155 and trial 4 *P*=0.312). Nevertheless, NKA activity of smolts in seawater was significantly higher than in other trials (trial 2 *P*=0.001, trial 3 *P*=0.045 and trial 4 *P*=0.029). There was a similar pattern in brackish water (trial 1 *P*=0.047, trial 3 *P*=0.005 and trial 4 *P*=0.173).

**Table 5.**
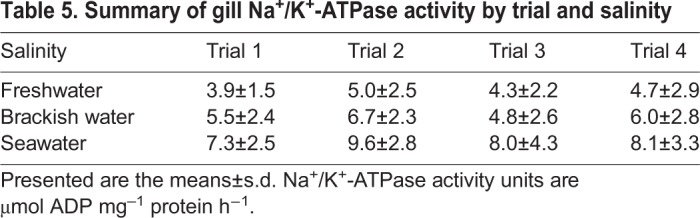
**Summary of gill Na^+^/K^+^-ATPase activity by trial and salinity**

### Relationship to physiological and body variables

Gene expression patterns with the largest separation for groups within treatments, i.e. salinity (PC2), temperature (PC1) and mortality (PC1) using live and distressed fish or live fish only, were generally not strongly correlated with body variables (Figs 2B,D,H, 3B,D; Table S3). However, salinity (PC2) had a significant positive correlation with both NKA activity and chloride concentration, as well as a negative correlation with relative startle response. Also, temperature (PC1) was positively correlated with both gill ventilation rate and NKA activity, whereas mortality (PC1) was positively correlated with many variables (i.e. plasma lactate and chloride concentrations, caudal fin yellowness and back roundness), and negatively correlated with plasma glucose concentration, body condition and thickness (Fig. 2H).

Gene expression patterns for DO (PC1 and PC2) using live and distressed fish or live fish only, in contrast to salinity, temperature and mortality, did not separate normoxia and hypoxia well, but there were correlations with body variables (Figs 2F, 3F; Table S3). For live and distressed fish, DO (PC1) was correlated with mortality, body condition, thickness, back roundness and caudal fin yellowness, as well as with gill ventilation rate, plasma lactate, glucose and chloride concentrations. For live fish only, there were fewer correlations with PC1, i.e. body condition, thickness, gill ventilation and plasma lactate concentration. Based on model selection directly comparing groups, fish exposed to hypoxia had higher gill ventilation rate (ANOVA, *P*<0.001) and anterior region brightness (*P*=0.039) than those exposed to normoxia.

## DISCUSSION

A laboratory challenge that exposed juvenile ocean-type Chinook salmon to multi-stressor conditions for 6 days identified gill gene expression biomarkers specific to salinity and temperature treatments, but not a DO treatment. Biomarker selection results using live and distressed (dead or moribund) fish were generally similar to those using live fish only for salinity and temperature, whereas half of the biomarkers were different for DO. For both analyses, these biomarkers showed a very high classification ability (at least 98%) for freshwater versus saline (brackish water and seawater) and for the three temperatures (10, 14 and 18°C). Although the DO biomarkers using live fish only had higher classification ability for normoxia than live and distressed fish (76% versus 60%), hypoxia was the same (75%) and there was a poor separation using PCA for both analyses. As expected, the 6-day test exposure revealed that most of the non-biopsied dead or moribund individuals were from seawater (88%) and were in the pre-smolt and de-smolt groups (98%). Importantly, we identified genes with expression associated with mortality across the 18 test groups (live versus dead or moribund). Here, we describe the changes in other physiological variables, body size, skin pigmentation and body morphology associated with smolt status, as well as the salinity, temperature and DO treatments.

### Smolt status effects

Juvenile fish placed into seawater outside of their smoltification physiological window as pre-smolts or de-smolts are known to experience high mortality (Björnsson et al., 2011; McCormick et al., 1998, 2013). Therefore, we anticipated that the four trials (March to August) would have a differential mortality among the non-biopsied fish, with the fish that were not optimally prepared (pre-smolt or de-smolt) experiencing a higher mortality in seawater than the prepared smolts. These results support other studies (e.g. Stich et al., 2015, 2016) that show that the degree of smoltification is important for seawater survival, and likely early marine survival. Consequently, physiological condition (related to smoltification stage) affects the severity of salinity, with mortality as a severely maladaptive or negative consequence (Schreck and Tort, 2016).

Smoltification typically involves silvering of the body, darkening of the caudal fin margins and elongation of the caudal peduncle, which are morphological changes that possibly improve camouflage and swim performance in marine habitats (Björnsson et al., 2011; McCormick et al., 1998). Correspondingly, the smolts in the present study had brighter anterior and posterior regions, darker caudal fins and longer caudal peduncles than pre-smolts; yet, the anterior region brightness decreased in de-smolts. Thus, common to smoltification in the present and a previous study (Houde et al., 2018 preprint) were increased caudal fin darkness and peduncle length, but changes in body brightness were not detected previously.

Although we detected significant differences for NKA activity between pre-smolt and smolts in freshwater, as may be expected (e.g. Kiilerich et al., 2007; Lemmetyinen et al., 2013; Piironen et al., 2013), differences between smolts and de-smolts were not detected. However, we can classify these freshwater individuals as pre-smolt, smolt and de-smolt using the candidate salinity genes (see Houde et al., 2018 preprint). Thus, our gene expression data may be a more sensitive indicator of smoltification and seawater preparedness than freshwater NKA activity. Similarly, a high NKA activity in freshwater prior to seawater entry may be unnecessary if a juvenile can rapidly increase NKA activity after it enters seawater during an appropriate physiological window (Bassett et al., 2018; Madsen and Naamansen, 1989). Even so, the NKA activity after 6 days in seawater was the highest in smolts compared with pre-smolts and de-smolts, which suggests that smolts can achieve a higher NKA activity in seawater than pre-smolts and de-smolts.

### Salinity biomarkers

The gene expression pattern (PC2) associated with the separation among salinity groups involved 10 or 11 biomarkers. In addition, the PC2 positive correlations with plasma chloride concentration and NKA activity are physiological indicators of salinity acclimation (Björnsson et al., 2011; McCormick et al., 1998). Fish in seawater must excrete excess ions primarily via the gills (Evans et al., 2005; Hwang and Lee, 2007). Therefore, it is of interest that two of the biomarkers were ion regulation genes (i.e. *CA4* and *CFTR-I*) with tight links to the positive end of PC2. Similarly, others have found a higher expression of ion regulation genes with transfer from freshwater to seawater (Flores and Shrimpton, 2012; Havird et al., 2013; Singer et al., 2002). Although another ion regulation gene, *NKAa1-b* (e.g. Björnsson et al., 2011; Nilsen et al., 2007), was significantly associated with smoltification and increased salinity, this gene will likely not be a good biomarker because it was not specific to salinity. *NKAa1-b* and NKA activity were secondarily influenced by temperature, as seen previously (Bassett et al., 2018). Regardless, at the other end of PC2, three genes were related to immunity (i.e. *CCL4*, *CCL19* and *IFI44*). Immunity genes may be suppressed during seawater transfer because of a proposed energetic trade-off between immunity and acclimation to seawater (Johansson et al., 2016; Makrinos and Bowden, 2016).

### Genes associated with mortality in seawater

Juveniles die in seawater because of internal ionic and osmotic disturbances, as detected by higher plasma ion concentrations (Blackburn and Clarke, 1987) and a low gill NKA activity (Kennedy et al., 2007; Stich et al., 2015, 2016). Correspondingly, the fish that died or became moribund shortly after seawater exposure in the present study had higher plasma chloride concentration, lower NKA activity, smaller body size (length, mass and elongation) and lower condition (including thickness) than fish surviving the 6-day test exposure. We also observed a lower startle response of fish in seawater compared with fish in freshwater and brackish water, which is consistent with Handeland et al. (1996), who observed a shorter predator escape distance and a higher piscine predation after seawater transfer. Although NKA activity and plasma chloride concentration in brackish water were higher than in freshwater, the few changes in mortality and body morphology in brackish water may be because the salinity was nearly isoosmotic to the fish, such that less energy would be needed for homeostasis compared with being in seawater (Morgan and Iwama, 1991; Stien et al., 2013; Webster and Dill, 2006).

Salinity (PC1) displayed a pattern with smolts at one extreme, pre-smolts and de-smolts in between, and mortality at the other extreme, for the 11 biomarkers using live and distressed fish. In the direction of mortality and away from smolts, juveniles had higher expression of two freshwater ion regulation genes (*NKAa1-a* and *PRLR*) and lower expression of a seawater ion regulation gene (*CFTR-I*). These results suggest that mortality in seawater is associated with a mismatch for ion regulation gene expression involved in internal ionic and osmotic regulation during transfer from freshwater to seawater.

### Temperature biomarkers

Five of the upregulated genes associated with the highest temperature (18°C) included genes encoding heat shock proteins (HSPs): two paralogues of *SERPINH* and two paralogues of *hsp90a* and *hsp70*. These genes are known to be the most frequent HSP genes to respond to high temperature among salmonids and other species (Akbarzadeh et al., 2018). The importance of molecular chaperoning of macromolecules during higher temperature is well established and includes an upregulation of many paralogues relating to HSP genes in adult sockeye salmon in response to chronic elevated temperature (Akbarzadeh et al., 2018; Jeffries et al., 2012, 2014b), such as *SERPIN*, *HSP70* and *HSP90a*. Indeed, HSP genes are upregulated in response to high temperature in more than 16 different fish species belonging to different taxa, including salmonids (Akbarzadeh et al., 2018). Therefore, the upregulation of HSP genes seems to be a robust temperature biomarker across all salmonids and likely many fish species.

In addition, the significant downregulation of two paralogues of *FKBP10* at the highest temperature compared with 10 and 14°C is an important discovery because downregulation of *FKBP10* was also observed previously in response to high temperature with adult sockeye salmon (Akbarzadeh et al., 2018; Jeffries et al., 2014b), catfish (Liu et al., 2013) and white sturgeon (Silvestre et al., 2010). Thus, differential expression of the *FKBP10* gene seems to be specific to a temperature challenge and could be a strong biomarker for chronic exposure to high temperature in fish. In addition, genes involved in protein biosynthesis, including *EEF2* (assays *ef2_14* and *ef2_3*) and *SFRS2*, were significantly downregulated in fish held in high temperature, which suggests that a decrease in protein biosynthesis may be a cellular energy-saving mechanism in response to high temperature (Jeffries et al., 2014b). In fact, previous studies have also shown that exposure to chronically elevated water temperature decreases the expression of *EEF2* and *SFRS2* genes in adult Pacific salmon (Akbarzadeh et al., 2018; Jeffries et al., 2012, 2014b).

Consistent with a higher metabolic rate and supportive convection mechanisms (Heath, 1973; Zhao et al., 2017), gill ventilation rate was higher at 14 and 18°C compared with 10°C. Also, plasma lactate concentration increased at high temperature, as seen previously with adult sockeye salmon (Jeffries et al., 2012; Steinhausen et al., 2008), which is an indication of inadequate oxygen delivery and increased reliance on anaerobic metabolism (Han et al., 2017; Pankhurst, 2011). NKA activity, which is an energy-consuming cell membrane ion pump (Mobasheri et al., 2000), was also higher in warm versus cold water, suggesting that enhanced NKA activity is a mechanism to cope with increased ion fluxes (Handeland et al., 2000; Vernberg and Silverton, 1979). Moreover, plasma glucose concentration decreased with higher temperatures, perhaps because the enhanced hepatic glycogen stores were being depleted with temperature (Chadwick and McCormick, 2017). Overall, these results of increased metabolism and energy utilization may help explain the smaller body size (length, mass and elongation) and lower condition (including thickness) of the juveniles kept in warmer temperatures. Similar changes in growth in response to higher temperature have been observed for juvenile Chinook salmon (Marine and Cech, 2004) and Arctic charr (Lyytikäinen et al., 2002).

### DO biomarkers

The nine or 11 DO (and temperature) biomarkers identified by gene shaving could not strongly separate normoxia and hypoxia. This may not be surprising given that the background candidate DO gene expression information for fish was limited, especially in salmonid species and for gill tissue. Currently, using the freshwater samples of the present study, we are undertaking an RNA-seq study to discover additional candidate DO genes, which will then be validated with other samples (unpublished data). Regardless, two genes (*HIF1A_6* and *COX6B1*) were primarily influenced by DO using model selection. *HIF1-*A is suggested as a reliable fish biomarker of hypoxia exposure (Wenger, 2002). The upregulation of *HIF1-A* in response to hypoxia has been also observed in Atlantic croaker (Rahman and Thomas, 2017), goby (Gracey et al., 2001), ruffe and flounder (Tiedke et al., 2014) and Pacific herring (Froehlich et al., 2015). *COX6B1* is a non-transmembrane subunit of COX, which could be upregulated in response to the higher oxidation during hypoxia. The upregulation of *COX6B1* has also been observed in both hypoxia (Long et al., 2015) and higher temperature (Garvin et al., 2015; Jeffries et al., 2012, 2014b).

Hypoxia alone did not influence juvenile mortality except during handling; however, the juveniles showed adaptive responses that modulated their behavioural and physical phenotype. Gill biopsied mortality was higher for hypoxia than normoxia in one of the de-smolt trials, but not the second de-smolt trial. It is possible that some gill filaments were damaged during juvenile handling, providing less efficient oxygen exchange in hypoxia and eventually causing mortality. Regarding behaviour, juveniles showed a higher ventilation rate for hypoxia compared with normoxia. One of the most evident physiological adjustments to hypoxia is increased ventilation in an effort to compensate for lower DO (Itazawa and Takeda, 1978; Steffensen et al., 1982). Juveniles in hypoxia also showed paler skin pigmentation compared with in normoxia. This result may be associated with the effects of skin pigmentation controlling hormones, including α-melanophore stimulating hormone (αMSH) and melanin concentrating hormone (MCH). These hormones are pleiotropic by not only controlling skin pigmentation, but also regulating the response to other stressors (Burton and Vokey, 2000).

### General mortality genes

Across the 18 test groups, both non-biopsied and biopsied dead or moribund fish upregulated seven genes associated with the physiological stress response. Changes in heat shock proteins (e.g. *hsp70_6* and *hsp90a_15* genes), metabolite (e.g. fructose-bisphosphate aldolase, *ALD_1*, and lactate, *LDH_1*, genes) and immune function (e.g. *FKBP5*, *RGS21* and *VEGFa_1* genes) are secondary in the stress response of fishes, after the primary release of stress hormones (Barton, 2002). Plasma lactate concentration was also higher for distressed (dead or moribund) fish in seawater.

Tertiary effects of stress can be the enhancement of disease susceptibility through the breakdown of immune barriers of defense (Aich et al., 2009; Marsland et al., 2002). The juveniles were screened for diseases by DFO prior to the commencement of our study. Although the juveniles passed the screening, this may not necessarily mean that the juveniles were not carrying agents that can cause disease. We examined the presence and loads of 47 salmon infectious agents in 79 distressed juveniles, and detected only two bacteria (i.e. *Candidatus Branchiomonas cysticola* and *Flavobacterium psychrophilum*) at elevated loads (within the range that can be associated with disease) in only a few individuals. Both bacteria are common in juvenile Chinook salmon, and their presence alone is not necessarily indicative of a disease state (Bass et al., 2017; Miller et al., 2017b; Tucker et al., 2018). If an outbreak of disease by either bacterium had occurred, and contributed to mortality, we would have expected a general elevation of the bacterium in most or all dying fish in the tank; instead, we observed only sporadic individuals with elevated levels of either bacterium among the tanks. These data suggest that there were no outbreaks of stress-induced disease during the 6-day test exposure, and instead the distressed juveniles of our study were the result of stress from seawater and gill biopsy.

### Conclusions

We identified gene expression biomarkers in salmonid gill tissue that were specific to salinity and temperature treatments across multi-stressor conditions, smolt status and mortality using a sophisticated experimental set-up. Similar biomarkers were not identified for DO. However, we are discovering and validating additional candidate dissolved oxygen biomarkers using RNA-seq on samples from the present study (unpublished data). We also identified genes associated with general mortality, with links to secondary protein products of the fish stress response. The changes in behaviour, plasma variables, NKA activity, body size, body morphology and skin pigmentation in response to the three stressors, as well as in response to mortality and smolt status, are also described. Most mortality occurred in seawater when juveniles were not optimally smolted and experienced osmotic and ionic disturbances, e.g. lower condition from dehydration and higher plasma ions. We highlight that moribund or dead juveniles may have a mismatch of the ion regulation gene expression patterns expected for seawater acclimation. Importantly, these salinity, temperature and, eventually, DO biomarkers can be used in natural environments to identify a specific stressor even under multi-stressor conditions.

## Supplementary Material

Supplementary information
