# Supplementary material for: Salmonid gene expression biomarkers indicative of physiological responses to changes in salinity and temperature, but not dissolved oxygen
Source: J Exp Biol. 2019 Jul 5;222(13):jeb198036. doi: 10.1242/jeb.198036 (PMC6633282; doi:10.1242/jeb.198036)
Supplement: Supplementary information [file jexbio-222-198036-s1.pdf]

Table S1. Summary of qPCR TaqMan assay designs and efficiencies for candidate dissolved oxygen genes

Presented are the forward (F), reverse (R), and probe sequences (P). Species abbreviations for efficieny: CK= Chinook salmon (*Oncorhynchus tshawytscha*), CO= Coho salmon (*O. kisutch*), CM= Chum salmon (*O. keta*), PK= Pink salmon (*O. gorbuscha*), SX= Sockeye salmon (*O. nerka*), AS= Atlantic salmon (*Salmo salar*), AC= Arctic charr (*Salvelinus alpinus*), RT= Rainbow trout (*O. mykiss*), and BT= Bull trout (*Salvelinus confluentus*).

Gene name	Assay name	Primers and TaqMan Probes	PCR Efficiency								
			CK	CO	CM	PK	SX	AS	AC	RT	BT
Myoglobin	MYO_1	F- GACTAGTTCTGAGCCGTCTGTTTG<br>R- CGATGCCTGCGAACTTAGG<br>P- AGAGCACCCAGAAAC	0.92	0.98	1.18	0.98	0.80	-	1.04	-	1.00
Hypoxia-inducible factor 1-alpha-like	HIF1A_6	F- AGAGGAGGCAGTGCTGTATTCAA<br>R- GGGACAAGGCCCTCCAAT<br>P- AGGGCCCTGACCATG	0.95	0.94	1.13	0.94	1.07	0.95	1.04	-	1.13
hypoxia inducible factor 1, alpha	HIF1A_7	F- TGGCAAATCTGCCTACGAATT<br>R- GCAGGCTCTTGGTCACATGA<br>P- ATCATGCCCTGGACTC	1.11	1.15	1.18	1.18	1.04	0.98	1.00	1.13	1.08
Insulin-like growth factor binding protein-like 1	IGFBP1	F- ACACAGCGGCGGATGTCT<br>R- CGCCATTTCAAACTTGTCGTT<br>P- ATGAACTGTAGGGTATATTGA	1.02	0.73	-	-	0.86	1.03	-	-	-
Insulin-like growth factor binding protein-1	IGFBP_10_v1	F- AGATAACCAGCTCTCAGCAGGAA<br>R- ATGTTTGTCACAGTTGGGTAGGTAGA<br>P- TAGGAGAGAAGTTCACCAAC	1.00	0.97	1.10	1.00	1.02	-	1.03	-	0.90
Fructose-bisphosphate aldolase A1	ALD_1	F- CGTGATTCAGTGTTGTCATCTTGA<br>R- TTCCTCCAGTGTTTTTTTCAGTCA<br>P- AAGTACATGTGCCTTCTT	0.99	0.94	1.46	1.39	0.89	1.04	1.25	1.05	0.79
Fructose-bisphosphate aldolase A1	ALD_4 _v1	F- GGGAGTGCCATGTCTTATTCG<br>R- TGTTGCAGAGGGTCGAGTAGAG<br>P- ACCTCCTAAGAAGCCG	1.33	-	-	1.10	1.03	1.45	1.00	1.08	0.94
L-lactate dehydrogenase B-A chain-like	LDH_1	F- GTCACTGCTCCCATTTTACACTCTAG<br>R- CCCAAACTCCCTCCCAGATAAC<br>P- CTGTTCTTAGCTTCCC	1.11	0.96	1.10	1.09	1.03	1.12	0.99	1.05	1.00
L-lactate dehydrogenase A chain-like	LDH_3	F- TTTGTTTAGTGTGTGCGAGAGTTG<br>R- TCCGTGCACTTACGGTTAGTTTT<br>P- CCAGAGCCATTCAGT	1.32	-	1.03	0.91	1.04	-	-	-	-
Solute carrier family 2, facilitated glucose transporter member 1	glu1	F- CTTCAAGGTGCCTGAGACCAA<br>R- CGAGTGCTTCTCTCCACCAGTAC<br>P- CCGCCAGTCGGCT	1.00	0.99	0.81	1.35	1.17	1.25	1.17	1.17	1.18

Gene name	Assay name	Primers and TaqMan Probes	PCR Efficiency								
			CK	CO	CM	PK	SX	AS	AC	RT	BT
Solute carrier family 2, facilitated glucose transporter member 2	glu2	F- GGAACCTTACATCAACTGGCTACA<br>R- GCAGTGGCCAGTAGTAGTCATTACC<br>P- CTGGTATACTACTGAGTCAGG	0.99	1.00	-	-	0.99	-	-	-	-
Phosphoglycerate kinase	PgK3_v1	F- GGCAAAGTGCTCCCTAAGTTTC<br>R- TAGAGAGCAGGGCTGGTGCTA<br>P- CACCCTGCGCTTGT	1.02	0.89	1.08	1.11	1.02	0.94	0.90	1.07	1.00
Phosphoglycerate kinase	PgK_5	F- TGGAGGCGTTTGTAGCTGAA<br>R- GAAACACAGCAGGAAGGAACATAA<br>P- CCACCCTCACATGCA	0.99	0.96	1.05	0.98	0.98	0.98	0.96	1.03	0.99
Enolase	Enolase_2	F- GTCTGAGCGTCTGGCTAAGTACAAC<br>R- CCAGCGAAAACAGCCTTGTC<br>P- AGCTGCTCAGGATTG	0.97	0.99	0.99	0.87	1.03	0.95	0.93	0.99	0.94
Glycogen phosphorylase	GlPh_1	F- CAGAACTGAAGGTCGCTTTCAA<br>R- CCCGTTTGTTTGCCACATTT<br>P- AGGCATCTCCATTTTA	0.98	0.91	0.99	0.89	1.00	0.97	0.93	1.7	0.99
Glycogen phosphorylase B	GlPh_4	F- CCTCTGCTTACTATTACTCCTGCTAGTCT<br>R- TGGGATTGGCATCTACAGTATACG<br>P- TGACTCCACCATGTGC	0.98	1.11	0.96	0.99	1.00	0.99	0.94	1.03	0.99
Hemoglobin (ch3)	HemA1_1	F- CCTTGTGACCCTGGCTATTCA<br>R- GCAAGGAATTTATCCACAGCAAT<br>P- CACTCCCGAAGTGCA	1.03	1.00	1.02	1.06	1.05	1.00	1.04	1.04	1.03
Heme oxygenase 1 (ch2)	HemOxi1_2	F- GGCGAGGGTCTGTCGTTCT<br>R- TCTACTCCTGTACAGCTGTTTGAACA<br>P- CCGGGCGTGAGCA	0.95	1.01	0.99	0.86	1.19	0.99	0.93	1.00	0.96
Heme oxygenase 1 (ch6)	HemOxi1_3	F- CTGCTGCAACACACAGATATACACA<br>R- GCCACCAGCAATTTAGGATTG<br>P- CTTCGCAAGATTGGTG	1.00	1.17	1.03	0.88	1.01	1.09	-	1.08	-
Heme oxygenase 2 (ch3)	HemOxi2_1	F- AGACACTCTCAGCCCCACAGA<br>R- GGGTGTTCTCCGCCTTCTC<br>P- CTTTCTGAACTGCTGGCT	0.95	0.99	0.96	0.99	1.00	1.03	0.95	0.97	0.96
Neuroglobin (ch1)	Ngb1_2	F- GCCTCTCCAGCCCTGAGTTC<br>R- GGAGGTTGTCCAGGTGACTGA<br>P- TGGACCATGTCACAAAG	0.94	1.06	0.98	1.00	1.14	-	0.90	1.04	1.01
Vascular endothelial growth factor A (ch5)	VEGFa_1	F- GGTCTGCTGTGGATATGAGTATCTTAAA<br>R- CCGTTGCACCTCTCAGTGAA<br>P- AGCGAAATTGTGACCATAA	0.97	1.01	0.93	0.76	1.00	-	0.90	0.93	-

Journal of Experimental Biology • Supplementary information

Table S2: Classification ability of different combinations of the groups using the identified treatments biomarkers.

A. Eighteen groups using live and distress fish

FW10N	FW10H	FW14N	FW14H	FW18N	FW18H	BW10N	BW10H	BW14N	BW14H	BW18N	BW18H
0.5714286	1.0000000	0.5555556	0.8571429	0.5000000	0.5000000	0.0000000	0.3333333	0.2000000	0.6250000	0.6666667	0.8000000

SW10N	SW10H	SW14N	SW14H	SW18N	SW18H
0.7142857	0.5000000	0.3333333	1.0000000	0.3333333	0.4375000

Average: 0.5515322

	FW10N	FW10H	FW14N	FW14H	FW18N	FW18H	BW10N	BW10H	BW14N	BW14H	BW18N	BW18H	SW10N	SW10H	SW14N	SW14H	SW18N	SW18H
FW10N	4	3	0	0	0	0	0	0	0	0	0	0	0	0	0	0	0	0
FW10H	0	6	0	0	0	0	0	0	0	0	0	0	0	0	0	0	0	0
FW14N	1	0	5	3	0	0	0	0	0	0	0	0	0	0	0	0	0	0
FW14H	0	0	1	6	0	0	0	0	0	0	0	0	0	0	0	0	0	0
FW18N	0	0	0	0	4	4	0	0	0	0	0	0	0	0	0	0	0	0
FW18H	0	0	0	0	3	3	0	0	0	0	0	0	0	0	0	0	0	0
BW10N	0	0	0	0	0	0	0	0	0	0	0	0	3	2	0	0	0	0
BW10H	0	0	0	0	0	0	1	2	0	0	0	0	1	2	0	0	0	0
BW14N	0	0	0	0	0	0	0	0	1	0	0	0	0	0	3	1	0	0
BW14H	0	0	0	0	0	0	0	0	1	5	0	0	0	0	1	1	0	0
BW18N	0	0	0	0	0	0	0	0	0	0	4	1	0	0	0	0	1	0
BW18H	0	0	0	0	0	0	0	0	0	0	1	4	0	0	0	0	0	0
SW10N	0	0	0	0	0	0	0	0	0	0	0	0	5	2	0	0	0	0
SW10H	0	1	0	0	0	0	0	0	0	0	0	0	5	6	0	0	0	0
SW14N	0	0	0	0	0	0	0	0	1	1	0	0	0	0	3	4	0	0
SW14H	0	0	0	0	0	0	0	0	0	0	0	0	0	0	0	13	0	0
SW18N	0	0	0	0	0	0	0	0	0	0	2	2	0	0	0	0	4	4
SW18H	0	0	0	0	1	0	0	0	0	0	2	3	0	0	0	0	3	7

B. Nine Groups (normoxia and hypoxia combined) using live and distress fish

FW10	FW14	FW18	BW10	BW14	BW18	SW10	SW14	SW18
1.0000000	0.9375000	1.0000000	0.2727273	0.5384615	0.9090909	0.9473684	0.9090909	0.6428571

Average: 0.7952329

	FW10	FW14	FW18	BW10	BW14	BW18	SW10	SW14	SW18
FW10	13	0	0	0	0	0	0	0	0
FW14	1	15	0	0	0	0	0	0	0
FW18	0	0	14	0	0	0	0	0	0
BW10	0	0	0	3	0	0	8	0	0
BW14	0	0	0	0	7	0	0	6	0
BW18	0	0	0	0	0	10	0	0	1
SW10	1	0	0	0	0	0	18	0	0
SW14	0	0	0	0	2	0	0	20	0
SW18	0	0	1	0	0	9	0	0	18

C. Six Groups (normoxia and hypoxia combined, and brackish and seawater combined) using live and distress fish

FW10	FW14	FW18	SW10	SW14	SW18
1.0000000	0.9375000	1.0000000	0.9666667	1.0000000	0.9743590

Average: 0.9797543

	FW10	FW14	FW18	SW10	SW14	SW18
FW10	13	0	0	0	0	0
FW14	1	15	0	0	0	0
FW18	0	0	14	0	0	0
SW10	1	0	0	29	0	0
SW14	0	0	0	0	35	0
SW18	0	0	1	0	0	38

Journal of Experimental Biology • Supplementary information

D. Eighteen Groups using live only fish

FW10N	FW10H	FW14N	FW14H	FW18N	FW18H	BW10N	BW10H	BW14N	BW14H	BW18N	BW18H
0.8000000	0.8000000	0.8333333	0.8333333	0.6000000	0.5000000	0.2000000	0.2000000	0.6000000	0.3333333	0.6666667	0.8000000

SW10N	SW10H	SW14N	SW14H	SW18N	SW18H
0.8000000	0.6250000	0.4000000	0.7500000	0.7142857	0.6250000

Average: 0.6156085

	FW10N	FW10H	FW14N	FW14H	FW18N	FW18H	BW10N	BW10H	BW14N	BW14H	BW18N	BW18H	SW10N	SW10H	SW14N	SW14H	SW18N	SW18H
FW10N	4	0	0	0	0	0	1	0	0	0	0	0	0	0	0	0	0	0
FW10H	1	4	0	0	0	0	0	0	0	0	0	0	0	0	0	0	0	0
FW14N	0	0	5	1	0	0	0	0	0	0	0	0	0	0	0	0	0	0
FW14H	0	0	1	5	0	0	0	0	0	0	0	0	0	0	0	0	0	0
FW18N	0	0	0	0	3	2	0	0	0	0	0	0	0	0	0	0	0	0
FW18H	0	0	0	0	3	3	0	0	0	0	0	0	0	0	0	0	0	0
BW10N	0	0	0	0	0	0	1	1	0	0	0	0	3	0	0	0	0	0
BW10H	0	0	0	0	0	0	1	1	0	0	0	0	1	2	0	0	0	0
BW14N	0	0	0	0	0	0	0	0	3	0	0	0	0	0	1	1	0	0
BW14H	0	0	0	0	0	0	0	0	2	2	0	0	0	0	0	2	0	0
BW18N	0	0	0	0	0	0	0	0	0	0	4	1	0	0	0	0	1	0
BW18H	0	0	0	0	0	0	0	0	0	0	1	4	0	0	0	0	0	0
SW10N	0	0	0	0	0	0	0	0	0	0	0	0	4	1	0	0	0	0
SW10H	0	0	0	0	0	0	0	1	0	0	0	0	2	5	0	0	0	0
SW14N	0	0	0	0	0	0	0	0	0	0	0	0	0	0	2	3	0	0
SW14H	0	0	0	0	0	0	0	0	1	0	0	0	0	0	1	6	0	0
SW18N	0	0	0	0	0	0	0	0	0	0	0	1	0	0	0	0	5	1
SW18H	0	0	0	0	0	0	0	0	0	0	0	3	0	0	0	0	0	5

E. Nine Groups (normoxia and hypoxia combined) using live only fish

FW10	FW14	FW18	BW10	BW14	BW18	SW10	SW14	SW18
1.0000000	0.9375000	1.0000000	0.2727273	0.5384615	0.9090909	0.9473684	0.9090909	0.6428571

Average: 0.7952329

	FW10	FW14	FW18	BW10	BW14	BW18	SW10	SW14	SW18
FW10	13	0	0	0	0	0	0	0	0
FW14	1	15	0	0	0	0	0	0	0
FW18	0	0	14	0	0	0	0	0	0
BW10	0	0	0	3	0	0	8	0	0
BW14	0	0	0	0	7	0	0	6	0
BW18	0	0	0	0	0	10	0	0	1
SW10	1	0	0	0	0	0	18	0	0
SW14	0	0	0	0	2	0	0	20	0
SW18	0	0	1	0	0	9	0	0	18

F. Six Groups (normoxia and hypoxia combined, and brackish and seawater combined) using live only fish

FW10	FW14	FW18	SW10	SW14	SW18
0.9	1.0	1.0	1.0	1.0	1.0

Average: 0.9833333

	FW10	FW14	FW18	SW10	SW14	SW18
FW10	9	0	0	1	0	0
FW14	0	12	0	0	0	0
FW18	0	0	11	0	0	0
SW10	0	0	0	23	0	0
SW14	0	0	0	0	24	0
SW18	0	0	0	0	0	26

Journal of Experimental Biology • Supplementary information

Table S3. Correlations between gene expression patterns and physiological, body size, skin pigmentation, and body morphology variables.

Displayed are the variable Pearson correlations (and *p*-value) with the genes expression patterns (PC1 and PC2) for salinity, temperature, dissolved oxygen, and mortality. Mortality correlations coded live individuals as 0 and moribund or dead individuals as 1. Relative startle response correlations are at the level of the 18 groups, which coded freshwater as 3, brackish as 2, and seawater as 1. Gill ventilation correlations are also at the level of the groups; gill ventilation values were corrected for initial trial differences and are the mean for trials 1, 3, and 4 combined; there was no video to analyze for trial 2.

variable	salinity		temperature		dissolved oxygen		mortality	
	PC1	PC2	PC1	PC2	PC1	PC2	PC1	PC2
Live and distress								
Physiological								
mortality	0.63 (<0.001)	0.18 (<0.001)	-0.19 (<0.001)	-0.66 (<0.001)	0.80 (<0.001)	-0.05 (0.272)	0.90 (<0.001)	-0.00 (0.982)
relative startle response	0.23 (0.360)	-0.89 (<0.001)	0.11 (0.658)	0.48 (0.044)	-0.68 (0.002)	0.01 (0.979)	-0.58 (0.012)	-0.45 (0.059)
gill ventilation rate	-0.11 (0.659)	0.03 (0.913)	0.32 (0.200)	-0.28 (0.262)	0.39 (0.113)	-0.15 (0.547)	0.21 (0.412)	-0.02 (0.953)
Na^+/K^+-ATPase activity	-0.12 (0.010)	0.36 (<0.001)	0.10 (0.045)	0.02 (0.737)	-0.10 (0.049)	-0.18 (<0.001)	-0.10 (0.036)	-0.09 (0.050)
lactate concentrations	0.34 (<0.001)	0.02 (0.828)	0.11 (0.115)	-0.43 (<0.001)	0.39 (<0.001)	-0.28 (<0.001)	0.49 (<0.001)	-0.15 (0.034)
glucose concentrations	0.01 (0.941)	-0.02 (0.772)	-0.12 (0.091)	0.23 (0.001)	-0.33 (<0.001)	0.01 (0.909)	-0.29 (<0.001)	-0.24 (0.001)
chloride concentrations	0.11 (0.367)	0.37 (0.001)	0.05 (0.685)	-0.55 (<0.001)	0.48 (<0.001)	-0.30 (0.011)	0.42 (<0.001)	0.07 (0.571)
Body size								
length	0.19 (<0.001)	0.04 (0.419)	-0.03 (0.508)	0.09 (0.058)	-0.32 (<0.001)	-0.47 (<0.001)	-0.19 (<0.001)	-0.61 (<0.001)
mass	0.21 (<0.001)	0.00 (0.938)	-0.05 (0.280)	0.08 (0.078)	-0.30 (<0.001)	-0.42 (<0.001)	-0.18 (<0.001)	-0.57 (<0.001)
condition	-0.33 (<0.001)	-0.10 (0.032)	-0.05 (0.334)	0.40 (<0.001)	-0.42 (<0.001)	0.24 (<0.001)	-0.46 (<0.001)	0.12 (0.012)
Skin pigmentation								
anterior brightness	0.02 (0.710)	-0.09 (0.083)	-0.01 (0.812)	0.00 (0.985)	0.06 (0.195)	0.09 (0.058)	0.06 (0.195)	0.05 (0.307)
caudal fin darkness	0.10 (0.050)	0.00 (0.929)	-0.05 (0.332)	-0.09 (0.063)	0.07 (0.167)	-0.10 (0.046)	0.10 (0.044)	-0.11 (0.032)
posterior brightness	0.12 (0.017)	0.01 (0.900)	0.03 (0.524)	0.02 (0.718)	-0.12 (0.020)	-0.17 (<0.001)	-0.06 (0.248)	-0.36 (<0.001)
caudal fin yellowness	0.01 (0.801)	-0.01 (0.906)	0.06 (0.223)	-0.14 (0.004)	0.31 (<0.001)	0.28 (<0.001)	0.24 (<0.001)	0.23 (<0.001)

Journal of Experimental Biology • Supplementary information

variable	salinity		temperature		dissolved oxygen		mortality	
	PC1	PC2	PC1	PC2	PC1	PC2	PC1	PC2
Body morphology								
elongation	0.06 (0.208)	0.05 (0.323)	-0.07 (0.170)	0.21 (<0.001)	-0.39 (<0.001)	-0.29 (<0.001)	-0.30 (<0.001)	-0.50 (<0.001)
back roundness	0.22 (<0.001)	0.00 (0.947)	0.03 (0.566)	-0.36 (<0.001)	0.38 (<0.001)	0.02 (0.714)	0.36 (<0.001)	0.02 (0.653)
caudal peduncle length	-0.07 (0.150)	-0.06 (0.204)	0.08 (0.104)	0.00 (0.948)	0.07 (0.174)	0.08 (0.107)	0.02 (0.636)	0.14 (0.006)
thickness	-0.46 (<0.001)	-0.16 (0.001)	0.04 (0.446)	0.42 (<0.001)	-0.49 (<0.001)	0.15 (0.003)	-0.54 (<0.001)	0.10 (0.043)
Live only								
Physiological								
relative startle response	-0.54 (0.020)	0.93 (<0.001)	0.03 (0.906)	-0.24 (0.328)	-0.30 (0.221)	-0.25 (0.320)		
gill ventilation rate	0.23 (0.348)	-0.07 (0.769)	0.32 (0.189)	-0.11 (0.656)	0.32 (0.193)	-0.14 (0.566)		
Na^+/K^+-ATPase activity	0.12 (0.036)	-0.40 (<0.001)	0.11 (0.046)	-0.03 (0.658)	0.08 (0.158)	-0.01 (0.813)		
lactate concentrations	-0.20 (0.015)	0.18 (0.025)	0.22 (0.007)	0.05 (0.536)	0.18 (0.027)	-0.15 (0.060)		
glucose concentrations	-0.26 (0.001)	0.06 (0.463)	-0.16 (0.057)	0.13 (0.107)	-0.04 (0.633)	0.07 (0.391)		
chloride concentrations	0.00 (0.985)	-0.40 (0.003)	0.05 (0.746)	-0.05 (0.734)	0.19 (0.175)	0.19 (0.165)		
Body size								
length	-0.67 (<0.001)	-0.37 (<0.001)	0.03 (0.619)	-0.02 (0.658)	0.15 (0.006)	0.20 (<0.001)		
mass	-0.64 (<0.001)	-0.33 (<0.001)	0.01 (0.897)	0.03 (0.592)	0.18 (0.001)	0.22 (<0.001)		
condition	0.26 (<0.001)	-0.04 (0.533)	-0.17 (0.003)	0.03 (0.566)	-0.18 (0.002)	-0.03 (0.633)		
Skin pigmentation								
anterior brightness	0.00 (0.995)	0.00 (1)	-0.02 (0.744)	-0.08 (0.169)	-0.12 (0.033)	-0.05 (0.375)		
caudal fin darkness	-0.12 (0.034)	-0.11 (0.052)	-0.07 (0.241)	-0.11 (0.058)	-0.07 (0.247)	0.12 (0.041)		
posterior brightness	-0.40 (<0.001)	-0.29 (<0.001)	0.09 (0.124)	-0.07 (0.222)	0.04 (0.462)	-0.01 (0.911)		
caudal fin yellowness	0.31 (<0.001)	0.01 (0.812)	0.12 (0.041)	-0.03 (0.617)	-0.07 (0.253)	-0.19 (0.001)		
Body morphology								
elongation	-0.56 (<0.001)	-0.35 (<0.001)	-0.06 (0.326)	-0.09 (0.128)	0.02 (0.722)	0.18 (0.001)		
back roundness	0.00 (0.945)	0.05 (0.417)	0.15 (0.007)	0.15 (0.01)	0.19 (0.001)	-0.11 (0.047)		

variable	salinity		temperature		dissolved oxygen		mortality	
	PC1	PC2	PC1	PC2	PC1	PC2	PC1	PC2
caudal peduncle length	0.15 (0.007)	0.14 (0.012)	0.07 (0.203)	-0.11 (0.051)	-0.06 (0.275)	-0.09 (0.125)		
thickness	0.36 (<0.001)	0.06 (0.327)	-0.09 (0.132)	-0.06 (0.290)	-0.27 (<0.001)	-0.18 (0.001)		

Journal of Experimental Biology • Supplementary information
